# The Effect of Coating Density on Functional Properties of SiN_x_ Coated Implants

**DOI:** 10.3390/ma12203370

**Published:** 2019-10-15

**Authors:** Luimar Correa Filho, Susann Schmidt, Alejandro López, Mathilde Cogrel, Klaus Leifer, Håkan Engqvist, Hans Högberg, Cecilia Persson

**Affiliations:** 1 Division of Applied Materials Science, Department of Engineering Sciences, Uppsala University, 75121 Uppsala, Sweden; luimar.filho@angstrom.uu.se (L.C.F.); alejandro.lopez@angstrom.uu.se (A.L.); mathilde.cogrel@gmail.com (M.C.); klaus.Leifer@angstrom.uu.se (K.L.); hakan.engqvist@angstrom.uu.se (H.E.); 2 Thin Film Physics Division, Department of Physics, Chemistry and Biology (IFM), Linköping University, 58183 Linköping, Sweden; Susann.Schmidt@ionbond.com (S.S.); hans.hogberg@liu.se (H.H.)

**Keywords:** silicon nitride, coating, reactive high-power impulse magnetron sputtering, wear, joint replacements

## Abstract

Ceramic coatings may be applied onto metallic components of joint replacements for improved wear and corrosion resistance as well as enhanced biocompatibility, especially for metal-sensitive patients. Silicon nitride (SiN_x_) coatings have recently been developed for this purpose. To achieve a high coating density, necessary to secure a long-term performance, is however challenging, especially for sputter deposited SiN_x_ coatings, since these coatings are insulating. This study investigates the time-dependent performance of sputter-deposited SiN_x_ based coatings for joint applications. SiN_x_ coatings with a thickness in the range of 4.3–6.0 µm were deposited by reactive high power impulse magnetron sputtering onto flat discs as well as hip heads made of CoCrMo. SiN_x_ compositional analysis by X-ray photoelectron spectroscopy showed N/Si ratios between 0.8 and 1.0. Immersion of the flat disks in fetal bovine serum solution over time as well as short-term wear tests against ultra-high molecular weight polyethylene (UHMWPE) discs showed that a high coating density is required to inhibit tribocorrosion. Coatings that performed best in terms of chemical stability were deposited using a higher target power and process heating.

## 1. Introduction

In order to reduce metal ion release, while maintaining or improving the wear resistance, different ceramic coatings have been evaluated for the metallic parts of joint implants, and are used in knee implants in sensitive patients [1,2]. Examples of ceramic coatings that have been investigated include alumina (Al_2_O_3_), chromium nitrides (CrN and CrCN), titanium nitride (TiN), nanocrystalline diamond (NCD), diamond-like carbon (DLC), zirconium nitride (ZrN) and silicon nitride (Si_3_N_4_). Alumina coatings, deposited with an Al_3_Ti interlayer, have been found to present a higher hardness similar to that demonstrated by bulk alumina ceramic, and the use of interlayers can improve crack resistance and adhesion to the substrate [3,4,5]. CrN and CrCN have shown reduced wear compared to some DLC coatings, and when sliding against polyethylene the result was similar to that of a commonly used bulk ceramic [6,7,8,9,10]. TiN has a well-known high wear resistance and with demonstrated reduced metal ion release compared to CoCr [3,11,12,13,14]. Nanocrystalline diamond (NCD) has shown good results after millions of cycles in wear simulators [3,15,16,17]. DLC is a coating with high hardness and a low coefficient of friction due to self-lubricating characteristics that has been found to give a reduced metal ion release, but there is a concern of cytotoxicity due to release of graphite nanoparticles [3,18,19,20,21,22]. While ZrN is already in use in knee implants, with an estimated service life between 20–25 years [23,24], there is a lack of coatings showing consistently good results in hip joint replacements, which have a more challenging wear pattern. Si_3_N_4_ is a particularly promising material due to its ability to dissolve into biocompatible elements, while most other coatings would show very limited solubility, which is an issue once wear particles are produced. Alternatively, the other coatings would dissolve releasing metallic ions, which is one of the reasons for applying the coating in the first place (to reduce metal ion release). Si_3_N_4_, on the other hand, has shown bacteriostatic properties [25,26,27,28,29,30,31,32,33], and silicon may actually have a positive effect on bone metabolism [34]. Its application could for these reasons result in a considerable reduction of osteolysis [26,35,36,37]. However, a compromise in the dissolution rate needs to be found, to allow for coatings with an adequate longevity, while permitting dissolution of any small particles being released.

A previous study by Filho et al. [38] evaluated the wear resistance of SiN_x_ coatings deposited with 1- and 3-fold rotation (needed for future implant deposition) in a ball-on-disc system, in a hard-on-hard contact. While all coatings showed low wear rates against the Si_3_N_4_ balls, it was found that the three-fold rotation reduced the coating density, which could influence the performance of the coating in its targeted application. There is therefore a need to further investigate the effect of rotation and coating density on SiN_x_ coating performance, and particularly over time, and this was the aim of the current study. Coatings deposited by three-fold rotation were taken a step further, by being deposited also onto full three-dimensional implants. Coatings were assessed in terms of adhesion over time in solution, as well as in wear tests against polymeric materials, as this is the most common counter surface in joint implants.

## 2. Materials and Methods

### 2.1. Materials

CoCrMo flat discs [39], (Peter Brehm GmbH, Weisendorf, Germany) of 21.9 mm diameter and 5 mm thickness were used as substrates, as this is the most commonly used alloy for hip joint heads [40]. For the 3D set-up, uncoated and coated CoCrMo heads (Peter Brehm GmbH, Weisendorf, Germany) for hip implants were used [41], with 32 and 36 mm of diameter.

For the wear tests of the hip heads, ultra-high molecular weight polyethylene (UHMWPE) GUR 1020 discs with 39.8 mm of diameter and 5 mm thickness were used, as UHMWPE is one of the most commonly used polyethylenes in joint implants [40].

A reactive high power impulse magnetron sputtering (rHiPIMS) process was developed for the deposition of a CrN interlayer, followed by a top layer of SiN_x_ or SiCN on CoCrMo flat discs and CoCrMo heads for hip implants, based on an earlier study [42]. A base pressure of less than 2 MPa was achieved prior to deposition. Process conditions applied are listed in Table 1. Group names indicate the substrate shape (2D for flat discs and 3D for hip heads), one- or three-fold rotation, process heating and the average silicon target power in kW, e.g., 2D-1f-H0-P3.4 indicates a silicon nitride-based coating deposited onto a CoCrMo flat disc using one-fold rotation, with a process heating of 0 kW and 3.6 and 3.2 kW silicon target powers, resulting in an average target power of 3.4 kW. A P1.7 silicon target power represents 1.8 and 1.6 kW, i.e., an average target power of 1.7 kW.

### 2.2. Methods

#### 2.2.1. Composition

The chemical composition of the SiN_x_ coatings, deposited onto Si(001) substrates, was assessed by X-ray photoelectron spectroscopy (XPS) (Axis UltraDLD, Kratos Analytical, Manchester, UK). The X-ray source was a monochromatic Al(Kα) X-ray radiation (hν = 1486.6 eV). During acquisition, the pressure in the analysis chamber was less than 1 × 10^−7^ Pa. XPS survey and high-resolution core level spectra of Si2p, Si2s, Ar2p, N1s, C1s and O1s were recorded on as-received samples and after a sputter clean for 120 s with a 2000 eV Ar-ion beam with an incidence angle of 70° with respect to the surface normal. Low energy electrons generated from a flood-gun was applied for automatic charge compensation throughout the acquisition due to the low conductivity of the SiN_x_ coatings. After subtraction of a Shirley-type background the composition of the coating was extracted with elemental cross sections provided by Kratos Analytical. The quantification accuracy of XPS is typically around ±5% for elements <10 at.% and ±2–3% for elements >10 at.%. 

#### 2.2.2. Surface Roughness

Coated and uncoated materials were characterized by vertical scanning interferometry (WYKO NT-110, Vecco, Germany) using a field of view (FOV) of 1× and objective lens 10×. UHMWPE discs were characterized by an optical profilometer (ZYGO NexView, Middlefield, CT, USA). The arithmetic average from five measurements was calculated for later statistical analysis. 

#### 2.2.3. Scratch Resistance over Time in Solution

Seven discs, one for each 2D process, were soaked in 25 vol.% of FBS solution for a period of 1, 3 and 6 weeks. Between these time points, samples were cleaned and dried following a previously defined protocol [43]. Further characterization by a scratch test was performed, using three repetitions, with progressive loads from 0 to 100 N, a Rockwell C diamond with a tip radius of 200 µm and a scratch length of 5 mm. A loading rate of 120 N/min and a horizontal displacement rate of 6 mm/min were used. Evaluation was conducted in a light optical microscope at 20× according to ref. [44].

#### 2.2.4. Wear Resistance

A custom-made holder was used to perform reciprocal wear tests of the implant heads against UHMWPE discs. Three repetitions were performed for each head (three per size and coating type), by turning the implant head in the holder to start with a pristine area. Tests were performed at a frequency of 1 Hz and stroke length of 10 mm, for 10^4^ cycles. During the tests all materials were kept in a heated PTFE bath at a temperature of 37 ± 3 °C. In order to mimic body fluid, 25 vol.% fetal bovine serum was used (FBS, HyClone, EU approved, origin South America), complemented with 0.075 wt.% sodium azide (Sigma-Aldrich, St. Louis, MO, USA, S8032-25G) and 20.0 mM ethylene-diaminetetraacetic acid solution (EDTA, Sigma-Aldrich, 03690), according to ASTM F32-00 (2006). The contact pressure applied for full heads against the polymer discs was 9 MPa [45]. The polymer disc wear rate was estimated from the cross sectional area of the wear tracks based on Archard’s wear equation [46]. This was measured using a Nexview™ NX2 3D Optical Surface Profiler.

#### 2.2.5. Cross Section Characterization

A focused ion beam was used to obtain the cross sections (FIB; FEI Strata DB235, FEI, Hillsboro, OR, USA). Before milling, all coated samples were sputtered with (Au/Pd) for 30 s then deposited with a platinum layer (1 µm) to avoid Ga^+^ damage. Milling energy used was 7000 pA and 500 pA for final polishing both at 30 kV. For the 2D materials the cross sections were performed before and after the specified time points in solution (see section on scratch resistance over time in solution) and for 3D materials before and after wear tests (see section on wear resistance).

#### 2.2.6. Statistical Analysis

Analysis of variance (one-way ANOVA) was used to assess differences between groups. A Levene’s test was used to assess homogeneity of variances between groups. If significant, Welch’s robust ANOVA was used together with Tamhane’s post-hoc test to assess differences between groups. Otherwise Scheffe’s post-hoc test was used. A critical value of α = 0.05 was used to assess significance.

## 3. Results

### 3.1. Composition

The amount of material deposited (at.%) on the coatings, as measured by XPS, is shown in Table 2. Samples with one-fold rotation presented higher silicon and nitrogen contents, lower oxygen and carbon contents, and slightly higher N/Si ratios compared to three-fold rotation coatings.

### 3.2. Surface Roughness

The average surface roughness for all samples is shown in Table 3 and Table 4. 

All of the coated 2D and 3D samples showed roughness values <50 nm, in accordance with standard requirements for the application [47]. 

Before wear tests the UHMWPE discs were polished to achieve a surface roughness of <2 µm, as confirmed in Table 4 [47]. No statistically significant difference was found between the different UHMWPE discs.

### 3.3. Scratch Resistance over Time

**Figure 1:**
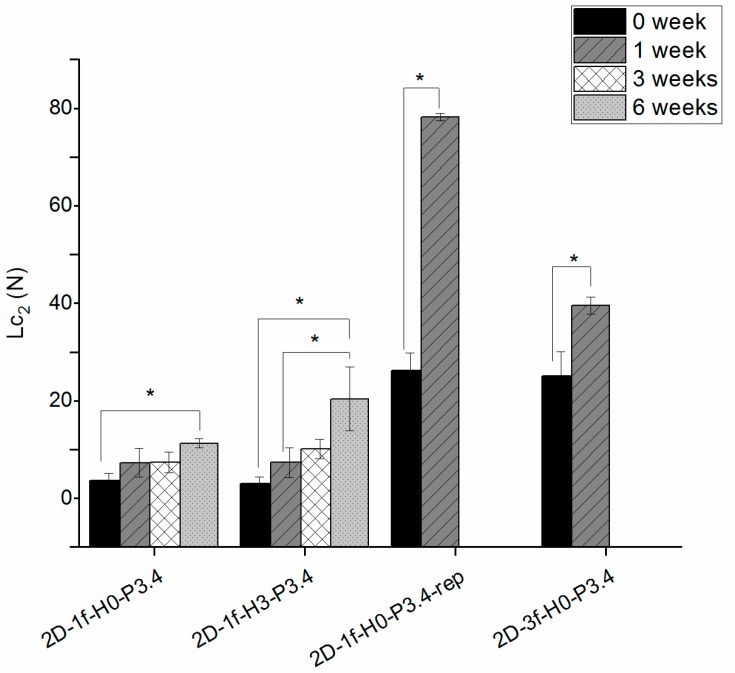
Critical load L_c2_ at different time points, from scratch tests on 2D coatings deposited on CoCrMo discs. * indicates a statistically significant difference between groups.

The scratch test tended to give increasing critical L_c2_ loads over time (Figure 1). These were statistically significant (*p* < 0.05) between week 0 and 6 for 2D-1f-H0-P3.4, between week 0 and 6 and between week 1 and 6 for 2D-1f-H3-P3.4. For coatings 2D-1f-H0-P3.4-rep and its three-fold version (2D-3f-H0-P3.4), there was a statistically significant difference between time points 0 and 1 weeks, but at 3 weeks these coatings had failed through dissolution and could not be tested. 

### 3.4. Wear Resistance

#### 3.4.1. Coefficient of Friction

**Figure 2:**
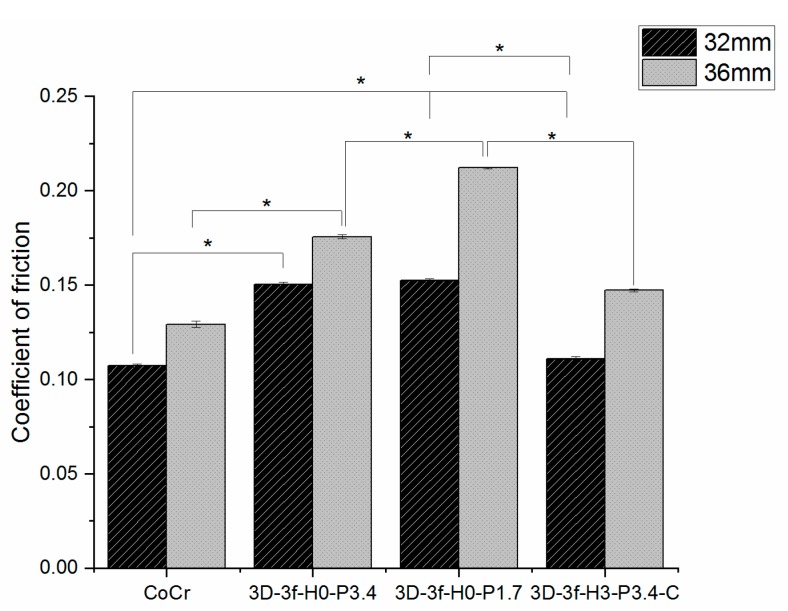
Coefficient of the friction of coated and non-coated 32 and 36 mm hip head implants during the reciprocal wear test against UHMWPE discs. * indicates a statistically significant difference between groups.

The coefficient of friction between uncoated and coated hip heads running against UHMWPE discs, as averaged between 2000–10,000 cycles, is shown in Figure 2. 

For the hip head size of 32 mm, statistical significance (*p* < 0.05) was found between uncoated CoCr and 3D-3f-H0-P3.4, uncoated CoCr and 3D-3f-H0-P1.7, 3D-3f-H0-P3.4 and 3D-3f-H3-P3.4-C and between 3D-3f-H0-P1.7 and 3D-3f-H3-P3.4-C.

The 36 mm head implants generally showed higher coefficient of friction values (Figure 2). The statistical analysis showed significance (*p* < 0.05) between uncoated CoCr and 3D-3f-H0-P3.4, between uncoated CoCr and 3D-3f-H0-P1.7 and between 3D-3f-H0-P1.7 and 3D-3f-H3-P3.4-C.

#### 3.4.2. Specific Wear Rate

**Figure 3:**
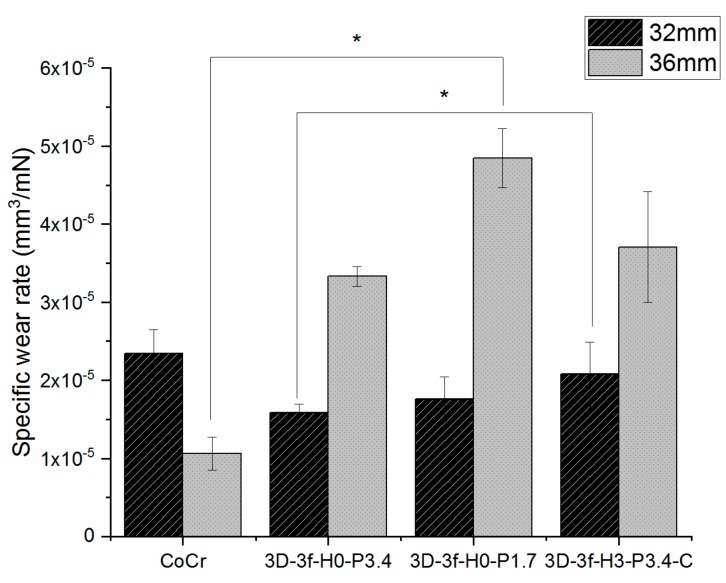
Specific wear rate of PE discs against 32 mm and 36 mm full head implants. * indicates a statistically significant difference between groups.

For the 32 mm heads, 3D-3f-H0-P3.4 and 3D-3f-H0-P1.7 showed a tendency for reduced specific wear rates compared to the uncoated samples (Figure 3). However, statistical significance (*p* < 0.05) was found only between 3D-3f-H0-P3.4 and 3D-3f-H3-P3.4-C for the 32 mm heads. 

For the 36 mm heads, the uncoated sample exhibited the lowest wear rate and 3D-3f-H0-P1.7 the highest specific wear rate. A statistically significant difference was found only between uncoated CoCr and 3D-3f-H0-P1.7 for the 36 mm heads.

### 3.5. Cross Sectional Morphology

**Figure 4:**
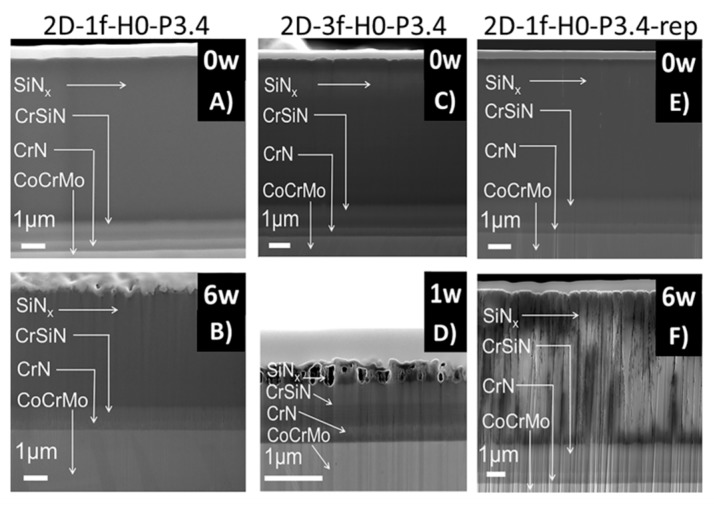
Cross sections from 2D coatings 2D-1f-H0-P3.4 (**a**,**b**), 2D-3f-H0-P3.4 (**c**,**d**) and 2D-1f-H0-P3.4-rep (**e**,**f**) on soaking time points of 0, 1 and 6 weeks.

**Figure 5:**
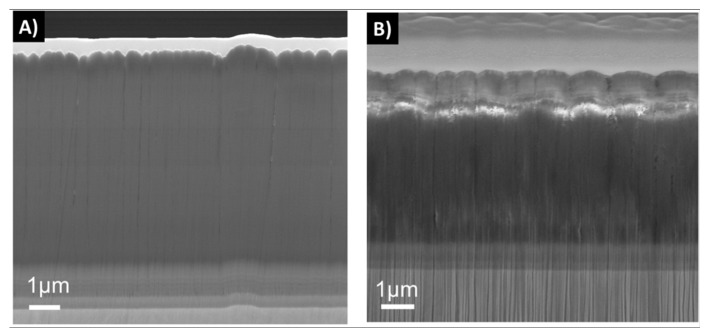
Cross sections from sample 3D-3f-H0-P3.4 before (**a**) and after (**b**) exposure to fetal bovine solution (FBS) showing surface morphology and coating layer cross sections.

**Figure 6:**
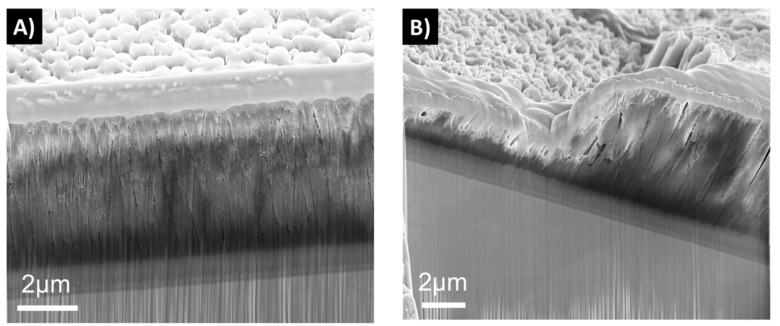
Cross sections from sample 3D-3f-H0-P1.7 before (**a**) and after the (**b**) reciprocal wear test, showing surface morphology and coating layer cross sections.

**Figure 7:**
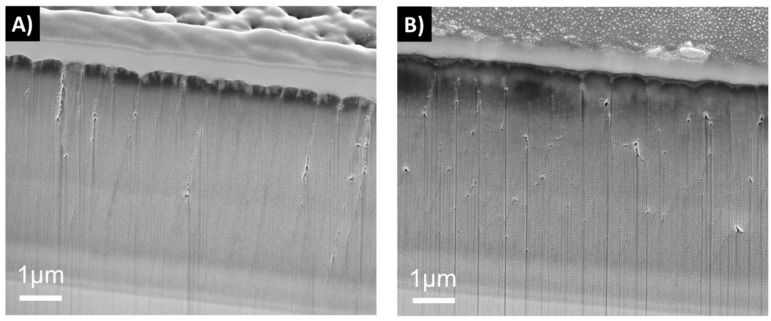
Cross sections from sample 3D-3f-H3-P3.4-C before (**a**) and after the (**b**) reciprocal wear test, showing surface morphology and coating layer cross sections.

The dissolution tests of the 2D samples showed that samples deposited with one-fold rotation gave denser coatings than the three-fold ones at the 0 week time point (Figure 4). After 6 weeks in FBS solution, an increased surface roughness was observed on all samples due to the dissolution process. Samples with three-fold rotation presented a rougher surface before exposure and failed through dissolution already at a time point of 1 week. In Figure 4, Figure 5, Figure 6 and Figure 7 vertical lines can be observed, starting from the small voids in the coating, which are related to tracks of the Ga^+^ ions and are therefore an artifact known as curtaining [48].

Cross sections were also made on the hip heads before and after the reciprocal wear tests against UHMWPE. A tribocorrosive process could be observed in the top layers, which was most severe for 3D-3f-H0-P1.7 (Figure 5, Figure 6 and Figure 7). Sample 3D-3f-H3-P3.4-C, which had a higher power in the process heating, as well as a SiCN top layer, showed the best resistance to the wear test (Figure 7).

## 4. Discussion

This work evaluated coating composition, adhesion, wear performance against UHMWPE and cross-sectional morphology as a function of soaking time and deposition parameters, including different rotation settings.

It has been found that when the N/Si ratio is ≥ 1.1, a stable, higher binding energy of Si–N results in an improved performance of these types of coatings in dissolution tests, in comparison to coatings with a N/Si ratio below 0.8 [49,50]. The coatings in this study showed a N/Si ratio between 0.8 and 1.0. The rotation applied during deposition plays an important role for the coating’s final morphology. Coatings with one-fold rotation exhibited denser morphologies (Figure 4, Figure 5, Figure 6 and Figure 7) due to the fact that sputtering is a line-of-sight deposition technique. Thus substrates deposited using one-fold rotation received a higher ad-atom flux and ion energies compared to those with three-fold rotation [51]. In other words, in three-fold rotation the substrate moved further away and closer to the plasma region influencing the plasma exposure time and deposition rate [52,53,54]. In this work, 2D coatings deposited with one-fold rotation, a high silicon target power and an N/Si ratio close to 1 (ranging from 0.98 to 1.03) showed higher coating densities compared to the 3D coatings (with a N/Si ratio ranging from 0.78 to 0.95). This was likely due to more Si–O bonds being formed [49,50] and three-fold rotation gave rise to lower density coatings resulting in increased porosity, which amplified the attachment of C and O when exposed to the air [42]. A further increase in Si target power or N content might improve the density and quality of these coatings.

The average surface roughness was comparable to the standard requirements and was achieved on both 2D and 3D coatings. However, a final polishing procedure would be needed for commercial purposes. 3D coatings had higher values for average surface roughness, which again can be explained by the increased rotation, as the substrate is bombarded with a lower and non-uniform material arrival rate [55].

SiN_x_ coating adhesion can be improved by the application of a CrN interlayer [55], which was also used in all coatings of this study. The less favorable performance of 2D-1f-H0-P3.4-rep in the scratch test over time, could be related to the higher O content of this coating, indicative of a lower density. Similarly, the generally less favorable performance of the 3D coatings without C was probably related to the deposition process (lower target power and process heating) resulting in less dense coatings, resulting from less flux of ion bombardment as well as less material being deposited [56,57], leading to a higher dissolution rate.

Despite the higher surface roughness of the coated samples compared to uncoated CoCr (Table 4), the coefficient of friction against the UHMWPE discs was in the same order of magnitude (Figure 2). Similarly for the specific wear rate of the UHMWPE discs (Figure 3). The coated 32 mm samples showed somewhat lower wear rate compared to the CoCr control, while the 36 mm samples showed a somewhat higher wear rate. Compared to previous studies on hard-on-soft contacts for joint implants, the specific wear rate results obtained were of a similar order of magnitude [58,59]. For example, Datta et al. [58] used a pin-on-disc system of UHMWPE pins running against TiN coatings at a contact pressure of 12 MPa in Hank’s solution at 37 °C, and found a specific wear rate of 1.9 ± 0.7 × 10^−5^ mm^3^/N·m. Berni et al. [59] ran stainless steel balls coated with yttria-stabilized zirconia against UHMWPE discs in a fetal bovine serum solution, and found a higher specific wear rate, of around 6 × 10^−4^ mm^3^/N·m, but also used a higher contact pressure of 30 MPa.

## 5. Conclusions

SiN_x_ coatings deposited by rHiPIMS with one- and three-fold rotation onto 2D and 3D CoCrMo substrates were evaluated. In summary, it was found that a high coating density was desirable to inhibit tribocorrosion. Coatings that performed best in terms of chemical stability were deposited using a higher target power and process heating.

## 6. Patents

Håkan Engqvist is co-inventor on a patent related to similar coatings.

## Figures and Tables

**Table 1:** SiN_x_ coating deposition parameters of process heating, substrate temperature, deposition pressure, N_2_/Ar flow ratio and resulting layer thicknesses.

Substrate	Group Name	Process Heating (kW)	Substrate Temperature (°C)	Deposition Pressure (MPa)	N_2_/Ar	SiN_x_ Thickness (nm)	Interlayer Thickness (nm)
CoCrMo discs	2D-1f-H0-P3.4	0	120	600	0.255	5385	1100
	2D-1f-H3-P3.4	3	300	600	0.255	5240	1030
	2D-1f-H0-P3.4-rep	0	120	600	0.26	6050	1700
	2D-3f-H0-P3.4	0	120	600	0.26	4399	1461
CoCrMo hip heads, 32 and 36 mm	3D-3f-H0-P3.4	0	120	600	0.28	4399	1461
	3D-3f-H0-P1.7	0	120	600	0.28	4492	1140
	3D-3f-H3-P3.4-C	3	300	600	0.28	4391	1169

**Table 2:** Results from X-ray photoelectron spectroscopy analysis for 2D and 3D coatings showing silicon, nitrogen, N/Si ratio, oxygen and carbon at.%. The quantification accuracy of XPS is typically around ±5% for elements <10 at.% and ±2%–3 % for elements >10 at.%.

Substrate	Process	Si (at.%)	N (at.%)	N/Si	O (at.%)	C (at.%)
CoCrMo disc	2D-1f-H0-P3.4	49.2	46.7	0.95	2.3	0.5
	2D-1f-H3-P3.4	47.4	48.8	1.03	1.7	0.8
	2D-1f-H0-P3.4-rep	48.49	44.19	0.91	6.51	0.81
	2D-3f-H0-P3.4	46.1	37.8	0.82	14.2	1.87
CoCrMo hip head 32 and 36 mm	3D-3f-H0-P3.4	46.1	37.8	0.82	14.2	1.87
	3D-3f-H0-P1.7	41.6	39.4	0.95	16.5	2.52
	3D-3f-H3-P3.4-C	40.6	31.5	0.78	12.2	15.7

**Table 3:** Surface roughness of 2D coatings on CoCr discs (average ± standard deviation).

Samples	Ra (nm)
2D-1f-H0-P3.4	15.6 ± 3.9
2D-1f-H3-P3.4	8.3 ± 0.6
2D-1f-H0-P3.4-rep	36.1 ± 15.5
2D-3f-H0-P3.4	8.9 ± 0.5

**Table 4:** Surface roughness of 3D coated, uncoated substrate and ultra-high molecular weight polyethylene (UHMWPE) discs used for reciprocal wear tests for 32 mm and 36 mm (average ± standard deviation).

Samples	Coated (3D) Implants		UHMWPE Discs	
	32 mm	36 mm	32 mm	36 mm
	Ra (nm)		Ra (µm)	
CoCr	11.7 ± 1.6	9.6 ± 1.8	1.1 ± 0.2	1.6 ± 0.4
3D-3f-H0-P3.4	39.9 ± 3.7	31.8 ± 1.8	0.8 ± 0.2	1.1 ± 0.5
3D-3f-H0-P1.7	43.1 ± 4.9	40.0 ± 5.5	0.7 ± 0.0	1.5 ± 0.3
3D-3f-H3-P3.4-C	29.5 ± 3.7	29.5 ± 3.5	1.1 ± 0.5	1.9 ± 0.2
